# Effects of *Trichoderma harzianum* Rifai and *Chaetomium cupreum* L.M. Ames on Biological Parameters of *Myzus persicae* (Sulzer) on Capia-Type Red Pepper (*Capsicum annuum* L.)

**DOI:** 10.3390/insects17030323

**Published:** 2026-03-17

**Authors:** Hilmi Kara

**Affiliations:** Department of Plant Protection, Faculty of Agriculture, Van Yüzüncü Yıl University, 65080 Van, Türkiye; hilmikara@yyu.edu.tr

**Keywords:** age-stage two-sex life table, biological control, *Myzus persicae*, *Capsicum annuum*, *Chaetomium cupreum*, *Trichoderma harzianum*

## Abstract

The green peach aphid, *Myzus persicae* (Sulzer), is a globally important agricultural pest whose management is increasingly challenged by widespread insecticide resistance, prompting interest in alternative and sustainable control strategies such as endophytic fungi. This study evaluated the effects of two endophytic fungi, *Trichoderma harzianum* and *Chaetomium cupreum*, applied individually or as a 1:1 mixture, on the population ecology of *M. persicae* feeding on capia-type red pepper (*Capsicum annuum* L.). Aphid development, survival, and reproduction were assessed using age-stage, two-sex life table analysis. Contrary to expectations, *T. harzianum* significantly enhanced aphid population growth, resulting in a higher intrinsic rate of increase (*r* = 0.42 d^−1^), finite rate of increase (*λ* = 1.52 d^−1^), and net reproductive rate (*R*_0_ = 87.67 offspring) compared to the Control (r = 0.32 d^−1^, *λ* = 1.37 d^−1^, *R*_0_ = 42.90 offspring). The Mixture treatment also increased population parameters, whereas *C. cupreum* showed limited effects on aphid life table traits. Population projections indicated that *T. harzianum* treatment could produce aphid populations approximately 380 times larger than the Control after 60 days. These results suggest that *T. harzianum* may improve host plant quality in ways that indirectly favor *M. persicae*. The findings highlight the importance of evaluating plant–fungus–herbivore interactions before incorporating endophytic fungi into integrated pest management programs.

## Simple Summary

The green peach aphid is a small sap-feeding insect that causes serious damage to many crops and is difficult to control because it has become resistant to many insecticides. Endophytic fungi, which live inside plant tissues without causing disease, are considered a promising alternative for sustainable pest control. In this study, we tested how two such fungi affect aphid populations when they colonize pepper plants. Surprisingly, *Trichoderma harzianum* greatly increased aphid survival and reproduction instead of reducing it, leading to much faster population growth compared to untreated plants. When both fungi were applied together, aphid numbers also increased, while *Chaetomium cupreum* alone had only minor effects. Long-term projections showed that aphid populations could become hundreds of times larger on plants treated with the growth-promoting fungus. These results show that beneficial microorganisms do not always suppress pests and can sometimes make plants more favorable to them. Our findings highlight the importance of carefully testing plant–fungus–insect interactions before using endophytic fungi in sustainable pest management programs, in order to avoid unintended consequences for farmers and food production.

## 1. Introduction

The green peach aphid, *Myzus persicae* (Sulzer, 1776) (Hemiptera: Aphididae), is a major agricultural pest worldwide, damaging many crops, including capia-type red pepper (*Capsicum annuum* L.). This polyphagous species feeds on over 400 plant species in more than 40 families, causing damage in two ways: direct feeding on phloem sap disrupts plant physiology and stunts growth, while indirect damage comes from transmitting over 100 plant viruses [1,2]. In pepper cultivation systems, *M. persicae* poses a particularly serious threat due to the crop’s economic importance and susceptibility to aphid-vectored viral diseases [3,4].

Conventional *M. persicae* control relies heavily on synthetic insecticides, but this approach faces serious problems: insecticide resistance, harm to pollinators and natural enemies, environmental contamination, and human health risks [2,5]. This has increased interest in sustainable, ecologically-based alternatives for aphid management within integrated pest management (IPM) frameworks. Biological control with beneficial microorganisms, especially fungal endophytes, has attracted interest because they act through multiple mechanisms and work well with organic systems [6,7]. These fungi colonize plant tissues without causing symptoms, often forming mutualistic relationships that improve plant growth, nutrient uptake, and stress tolerance. Some species also produce secondary metabolites with insecticidal activity or trigger systemic resistance, potentially reducing herbivore performance [8]. Among endophytic fungi, species from the genus *Trichoderma* and *Chaetomium* species have received particular attention for biocontrol and growth promotion. *Trichoderma* spp. are cosmopolitan soil fungi that suppress plant pathogens via mycoparasitism, antibiosis, resource competition, and induced resistance [9]. *T. harzianum*, in particular, has been extensively commercialized as a biofungicide and is known to enhance plant growth by solubilizing nutrients, producing plant hormone-like compounds, and activating plant defense pathways [10,11].

Similarly, *Chaetomium* species, particularly *C. cupreum* and *C. globosum*, have demonstrated effectiveness as biological control agents against various soil-borne pathogens and have shown antibiotic activity against nematodes and some insect pests [12]. These fungi produce diverse bioactive secondary metabolites, including chaetoglobosins, azaphilones, and various alkaloids that exhibit antimicrobial and insecticidal properties. Both *Trichoderma* and *Chaetomium* species are capable of colonizing plant roots endophytically and modulating plant physiology and chemistry in ways that may influence plant-herbivore interactions.

Endophyte–herbivore interactions vary widely with fungal strain, host plant, insect species, and environment [13]. Many studies show reduced herbivore performance through induced resistance, altered nutrition, or deterrent compounds [14,15], but recent work shows unexpected results. Some *Trichoderma* and *Metarhizium* isolates actually increase aphid populations, possibly by boosting plant nitrogen uptake and improving nutritional quality for herbivores [13]. This paradox shows why specific fungal isolates must be carefully tested before biocontrol use.

Despite widespread use of *Trichoderma* and *Chaetomium* for disease control and growth promotion, plant-mediated effects on phloem-feeding insects remain poorly characterized and appear highly dependent on fungal strain and plant genotype.

Most studies examining *Trichoderma*-insect interactions have focused on entomopathogenic effects through direct contact or on chewing herbivores rather than phloem-feeding insects. Recent evidence has revealed striking cultivar-specific and strain-specific responses: *T. harzianum* showed no significant effect on *M. persicae* populations on Demre-type pepper cultivar [16]. Conversely, Kara [17] reported that *T. harzianum* significantly reduced the intrinsic rate of increase (*r* = 0.332 d^−1^) and fecundity of *M. persicae* on Kasırga F1 bell pepper cultivar, while *T. asperellum* significantly increased aphid populations on Mazamort three-lobed pepper variety [18]. Furthermore, while *Chaetomium* species have been studied for their effects on some insect pests, their impact on aphid biology and population dynamics has received limited attention in the scientific literature.

The age-stage, two-sex life table approach [19,20] provides a comprehensive framework for assessing the population-level effects of treatments on insects by incorporating stage differentiation and male individuals, addressing the limitations of traditional female-only life tables [21]. This methodology allows for the calculation of critical population parameters, including the intrinsic rate of increase (*r*), net reproductive rate (*R*_0_), finite rate of increase (*λ*), and mean generation time (*T*), which together provide insights into population growth potential and the long-term impacts of control interventions. Additionally, population projection models based on life table data can forecast future population dynamics and help predict the efficacy of management strategies over extended time periods.

It was evaluated how two endophytic fungi (*Trichoderma harzianum* and *Chaetomium cupreum*), applied individually or in combination (1:1 mixture), affected the life history and population dynamics of *M. persicae* on capia-type red pepper. It was hypothesized that fungal colonization would alter plant physiology and suppress aphid performance. Using age-stage, two-sex life table analysis, we quantified effects on development, survival, longevity, and fecundity, then projected long-term population trends to assess their potential contribution to sustainable, environmentally friendly integrated pest management (IPM) strategies aimed at reducing reliance on chemical insecticides in pepper production systems.

## 2. Materials and Methods

### 2.1. Microorganisms

The fungal isolates of *Trichoderma harzianum* and *Chaetomium cupreum* were obtained from stock cultures maintained in the Mycology Laboratory of the Faculty of Agriculture at Van Yüzüncü Yıl University (Van, Türkiye). These isolates were obtained from agricultural soils in Van province, Türkiye, and identified by morphological and molecular methods. They have not been deposited in an international culture collection and lack formal accession numbers. Researchers needing these strains may contact the corresponding author. Depositing the isolates in a recognized collection (such as CBS, ATCC, or a national collection) would improve reproducibility and comparative studies. Future work should include formal strain deposition. Spore suspensions were prepared from colonies cultured on potato dextrose agar (PDA; Merck Ltd., Darmstadt, Germany) for one week, and a concentration of 1 × 10^8^ spores/mL was achieved using a hemocytometer. For the Mixture treatment, spore suspensions of both fungi were combined in equal volumes (1:1, *v*/*v*) to obtain a final concentration of 1 × 10^8^ spores/mL. Although *T. harzianum* and *C. cupreum* are commonly reported as endophytic fungi capable of colonizing plant roots, endophytic colonization was not directly confirmed in this study. The observed effects on aphid performance may be due to root zone colonization and plant-mediated alterations rather than confirmed endophytic settlement. Future studies should include direct confirmation of endophytic colonization through tissue isolation and molecular validation.

### 2.2. Plant Material

The pepper (*Capsicum annuum* L.) variety utilized in this investigation was Avşar F1, a capia-type red pepper cultivar (Multi Seed Company, Antalya, Türkiye). This commercially important variety is extensively cultivated and widely preferred in pepper production systems. The fruits of this cultivar are characterized by their length of 10–12 cm, crisp texture, sweet flavor profile, uniform morphological structure, and distinctive dark green coloration. This variety is primarily intended for fresh market consumption.

### 2.3. Insect

*Myzus persicae* colony used in this study was sourced from individuals collected in May 2024 from pepper fields in Van province, Türkiye, and subsequently maintained for over 10 generations on the Avşar F1 pepper variety at the Plant Protection Laboratory of Van Yüzüncü Yıl University prior to the experiments. The colony was maintained under controlled conditions (25 ± 2 °C, 65 ± 5% RH, 16:8 h light:dark) without exposure to insecticides.

### 2.4. Experimental Design

The experimental design consisted of four treatments: *T. harzianum*, *C. cupreum*, a mixture of both fungi, and a control group. Pepper seedlings at the 4–5 true leaf stage (approximately 3–4 weeks after germination) were carefully removed from nursery trays, and soil particles on roots were gently washed away with tap water. Roots were then dipped in relevant fungal spore suspensions (1 × 10^8^ spores/mL) or sterile distilled water (Control) for 2–3 min at room temperature (25 ± 2 °C). After the treatment, the seedlings were immediately planted in 4-L plastic pots (filled with a 2:1, *v*/*v* ratio sterile peat-perlite mixture). Experiments were started when the plants reached approximately 4–6 weeks of age.

At aphid introduction, plants had 6–8 true leaves and showed uniform growth across treatments. All plants appeared healthy with no visible disease, nutrient deficiency, or stress symptoms throughout the experiment. Plants were kept at 25 ± 2 °C, 65 ± 5% RH, 16:8 h L:D, and watered as needed.

Thirty replicates were established for each treatment, with one newly-born aphid nymph (<12 h old) confined per plant using a clip cage (approximately 2 cm diameter × 2 cm height) attached to a fully expanded leaf on the middle section of the plant.

### 2.5. Life Table

Newly born nymphs of *Myzus persicae* from the stock colony were individually confined in leaf cages (approximately 2 cm diameter × 2 cm height). The upper opening was covered with fine mesh to prevent escape while allowing ventilation. Individuals were checked daily to record moults and development time. After adult emergence, the number of nymphs produced each day was recorded, and newborns were removed immediately to avoid crowding and to ensure accurate fecundity counts. Observations continued until all individuals died. All experiments were conducted at 25 ± 2 °C, 65 ± 5% relative humidity, and a 16:8 h (light:dark) photoperiod.

### 2.6. Statistical Analysis

Life table parameters were analyzed according to the age-stage, two-sex life table theory [19,20]. Population parameters, including the net reproductive rate (*R*_0_), intrinsic rate of increase (*r*), finite rate of increase (*λ*), mean generation time (*T*), age-specific survival rate (*l_x_*), age-stage-specific survival rate (*s_xj_*), age-specific fecundity (*m_x_*), age-specific net maternity (*l_x_m_x_*), life expectancy (*e_xj_*), and reproductive value (*v_xj_*), were calculated using the TWOSEX-MSChart software (Table 1) [22,23,24,25]. Variances and standard errors of the parameters were estimated using the bootstrap technique (100,000 replicates) based on the principles of set theory and the multinomial theorem as described by Chi et al. (2022) [26]. Differences among treatments were evaluated using the paired bootstrap test at a significance level of *p* < 0.05.

### 2.7. Population Projection

To forecast population growth and stage structure under each treatment, life table data were used to project population dynamics over 60 days. Projections were initiated with a cohort of 10 newly born nymphs and simulated using the TIMING-MSChart program [28]. The program uses age-stage, two-sex life table data (stage-specific survival and fecundity) to estimate total population size and the numbers of individuals in each stage (nymphal instars and adults) for each day of the projection period.

The TIMING-MSChart program uses demographic parameters (*r*, *λ*, *R*_0_) derived from the complete life table data (*n* = 30 per treatment) rather than the starting cohort size, ensuring accurate population predictions [19,20,24,28]. Chi [19] demonstrated that population projections based on age-stage, two-sex life table theory accurately predict stage structure and growth dynamics regardless of initial population size.

## 3. Results

### 3.1. Development, Longevity and Fecundity of Myzus persicae

The development, longevity, and fecundity of *Myzus persicae* reared on pepper plants treated with *Trichoderma harzianum*, *Chaetomium cupreum*, their Mixture, and the Control are summarized in Table 2. In all treatments, females began reproducing on the first day of adulthood (adult pre-reproductive period, APRP = 0), so the preadult duration and the total pre-reproductive period were effectively identical; therefore, the latter is not reported separately.

Variable effects among treatment groups were observed in the duration of nymphal stages. Variable effects were observed among treatments in individual nymphal instar durations (Table 2). The N1 instar was significantly longer in *C. cupreum* (1.93 ± 0.08 days) compared to the Control (1.50 ± 0.09 days), while *T. harzianum* and the Mixture were intermediate. All treatments showed similar N2 duration (1.07–1.23 days). For N3, *C. cupreum* (1.57 ± 0.09 days) was longer than the Mixture (1.30 ± 0.08 days). During the N4 instar, Control (2.13 ± 0.15 days) and Mixture (2.03 ± 0.14 days) groups exhibited significantly longer durations than *C. cupreum* (1.47 ± 0.09 days) (*P_CCc_* = 0.00025; *P_MCc_* = 0.00091), while *T. harzianum* (1.77 ± 0.13 days) showed intermediate duration (*p* > 0.05). Despite these differences in individual instars, total preadult developmental time did not differ among treatments (*p* > 0.05), ranging from 6.13 to 6.20 days. Although treatments altered individual instar durations, total development time remained similar, likely due to compensation effects.

Significant differences were observed among treatment groups in adult longevity duration (*p* < 0.05). The Mixture treatment exhibited the longest adult longevity (28.73 ± 1.15 days), which was significantly longer than *C. cupreum* treatment (25.77 ± 0.94 days) (*P_MCc_* = 0.04534), while *T. harzianum* (26.90 ± 1.03 days) and Control (25.93 ± 1.53 days) showed intermediate values with no significant differences from other treatments (*p* > 0.05). Total longevity followed the same pattern as adult longevity, with Mixture (34.87 ± 1.14 days) significantly exceeding *C. cupreum* (31.97 ± 0.92 days) (*P_MCc_* = 0.04825), while *T. harzianum* (33.03 ± 1.03 days) and Control (32.13 ± 1.47 days) exhibited intermediate durations (*p* > 0.05).

No statistically significant differences were found among treatment groups for total preoviposition period (TPRP) (*p* > 0.05). Control and *C. cupreum* treatments showed identical values (6.20 ± 0.14 and 6.20 ± 0.07 days, respectively), while *T. harzianum* (6.13 ± 0.12 days) and Mixture (6.13 ± 0.11 days) also exhibited equal durations. All pairwise comparisons revealed *p*-values ranging from 0.6196 to 1.0, indicating no significant differences among any treatments (*p* > 0.05).

There were significant variations among treatment groups in the oviposition period (*p* < 0.05). Mixture treatment exhibited the longest oviposition period, which was significantly greater than *C. cupreum* (*P_MCc_* = 0.00346), while Control also showed significantly longer period compared to *C. cupreum* (*P_CCc_* = 0.03499). *T. harzianum* demonstrated intermediate values with no significant differences from other treatments (*p* > 0.05). Similarly, oviposition days showed significant differences among groups (*p* < 0.05), with Mixture (18.30 ± 1.30 days) exhibiting significantly more oviposition days than *C. cupreum* (14.17 ± 0.94 days) (*P_MCc_* = 0.00969), while Control (16.13 ± 1.12 days) and *T. harzianum* (15.73 ± 0.97 days) showed intermediate values (*p* > 0.05).

Fecundity differed significantly among treatments (Table 2). *T. harzianum* produced the highest values (87.67 ± 7.12 offspring), significantly exceeding all other treatments (*p* < 0.00001). Mixture (51.27 ± 3.38 offspring) also exceeded *C. cupreum* (38.00 ± 2.90 offspring), while Control (42.90 ± 3.44 offspring) showed intermediate values.

### 3.2. Population Parameters

Significant differences were found among treatment groups with respect to fundamental population parameters (*p* < 0.05) (Table 3). The intrinsic rate of increase (*r*) and finite rate of increase (*λ*) parameters exhibited parallel patterns across all treatments, reflecting the mathematical relationship between these two measures of population growth. The *T. harzianum* treatment demonstrated the highest population growth potential, with an intrinsic rate of increase of 0.42 ± 0.01 d^−1^ and a finite rate of increase of 1.52 ± 0.02. These values were significantly higher than all other treatments, including the Mixture (*r* = 0.34 ± 0.01 d^−1^; *λ* = 1.41 ± 0.01; *P_ThM_* < 0.00001), *C. cupreum* (*r* = 0.32 ± 0.01 d^−1^; *λ* = 1.38 ± 0.01; *P_ThCc_* < 0.00001), and Control (*r* = 0.32 ± 0.01 d^−1^; *λ* = 1.37 ± 0.01; *P_ThC_* < 0.00001) groups. Among the remaining treatments, the Mixture group exhibited significantly higher growth rates compared to the Control group (*P_MC_* = 0.01153 for *r*; *P_MC_* = 0.01117 for *λ*), while no significant difference was detected between the Mixture and *C. cupreum* groups (*P_MCc_* = 0.05911 for *r*; *P_MCc_* = 0.05893 for *λ*). The Control and *C. cupreum* groups displayed comparable population growth rates (*P_CCc_* = 0.40556 for *r*; *P_CCc_* = 0.40560 for *λ*).

Highly significant differences were found in net reproductive rate among treatment groups (*p* < 0.05). *Trichoderma harzianum* produced the highest *R*_0_ value (87.67 ± 7.12 offspring per individual), which was significantly greater than all other treatments: Mixture (51.27 ± 3.38 offspring) (*P_ThM_* < 0.00001), Control (42.90 ± 3.44 offspring) (*P_ThC_* < 0.00001), and *C. cupreum* (38.00 ± 2.90 offspring) (*P_ThCc_* < 0.00001). Mixture treatment exhibited significantly higher *R*_0_ compared to *C. cupreum* (*P_MCc_* = 0.00287), while no significant differences were detected between Mixture and Control (*P_MC_* = 0.08298) or between Control and *C. Cupreum* (*P_CCc_* = 0.27793).

The mean generation times were significantly different among treatment groups (*p* < 0.05). The control group exhibited the longest generation time (11.94 ± 0.28 days), which was significantly greater than *T. harzianum* (10.76 ± 0.29 days) (*P_CTh_* = 0.00397). Mixture (11.58 ± 0.25 days) also showed significantly longer generation time compared to *T. harzianum* (*P_MTh_* = 0.03712). No significant differences were found between Control and Mixture (*P_CM_* = 0.33011), Control and *C. cupreum* (11.25 ± 0.22 days) (*P_CCc_* = 0.05405), Mixture and *C. cupreum* (*P_MCc_* = 0.3346), or *C. cupreum* and *T. harzianum* (*P_CcTh_* = 0.19316).

Treatment groups exhibited highly significant differences in population doubling time (*p* < 0.05). *Trichoderma harzianum* demonstrated the shortest doubling time (1.67 ± 0.05 days), which was significantly lower than all other treatments: Control (2.20 ± 0.06 days) (*P_ThC_* < 0.00001), *C. cupreum* (2.14 ± 0.04 days) (*P_ThCc_* < 0.00001), and Mixture (2.04 ± 0.04 days) (*P_ThM_* < 0.00001). Control exhibited significantly longer doubling time compared to Mixture (*P_CM_* = 0.01478), while no significant differences were found between Control and *C. cupreum* (*P_CCc_* = 0.40406) or between *C. cupreum* and Mixture (*P_CcM_* = 0.06191).

Significant variations were found in gross reproductive rate among treatment groups (*p* < 0.05). *T. harzianum* produced the highest GRR value (116.01 ± 15.46 offspring), which was significantly greater than all other treatments: Mixture (64.76 ± 4.75 offspring) (*P_ThM_* = 0.00082), Control (59.10 ± 6.43 offspring) (*P_ThC_* = 0.00004), and *C. cupreum* (59.13 ± 11.63 offspring) (*P_ThCc_* = 0.00005). No significant differences were detected among Mixture, Control, and *C. cupreum* treatments (*P_MC_* = 0.28826; *P_MCc_* = 0.4243; *P_CCc_* = 0.8115).

**Figure 1:**
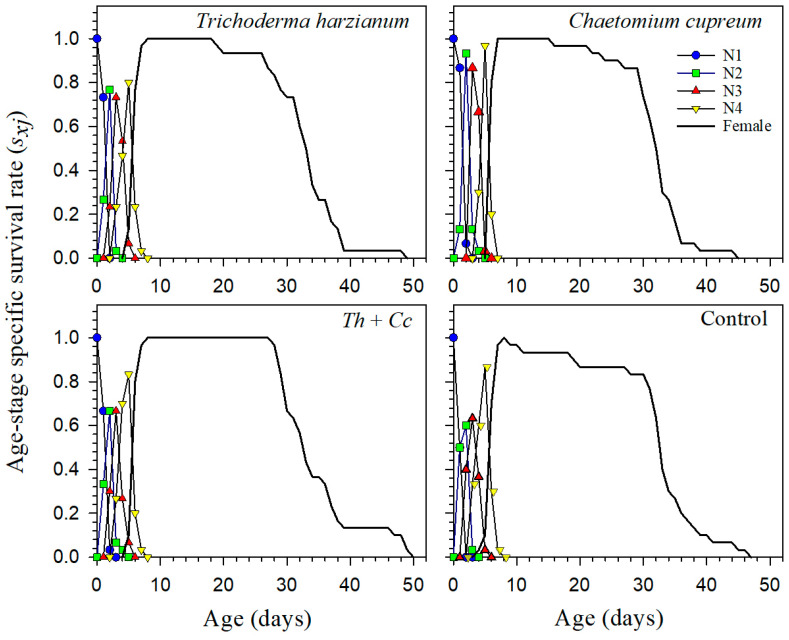
Age-stage specific survival rate (*s_xj_*) of *Myzus persicae* feeding on pepper plants treated with *Trichoderma harzianum* (*Th*), *Chaetomium cupreum* (*Cc*), their Mixture (*Th + Cc*), and Control (C).

**Figure 2:**
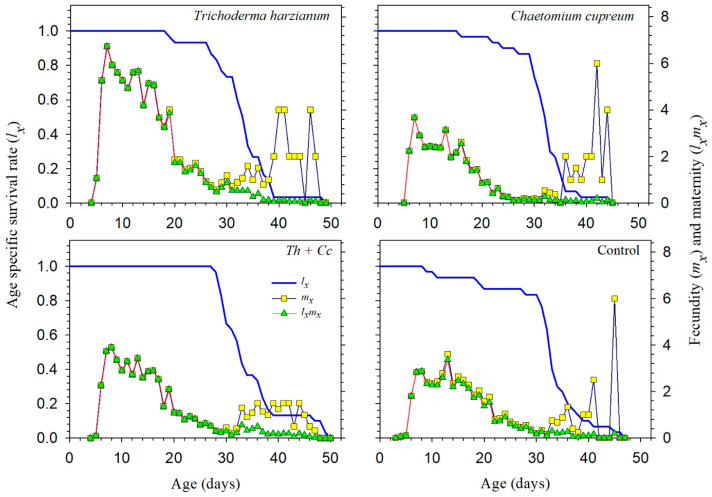
Age-specific survival rate (*l_x_*), fecundity (*m_x_*) and maternity (*l_x_m_x_*) of *Myzus persicae* feeding on pepper plants treated with *Trichoderma harzianum* (*Th*), *Chaetomium cupreum* (*Cc*), their Mixture (*Th + Cc*), and Control (C).

The age-stage specific survival (*s_xj_*) and age-specific survival (*l_x_*) curves showed overlapping stage distributions in all treatments, reflecting normal variability in aphid development (Figure 1). Adult survival persisted longer in the *T. harzianum* and Mixture treatments than in the Control and *C. cupreum* treatments, which is consistent with their longer longevity and higher population growth rates. Age-specific fecundity (*m_x_*) and maternity (*l_x_m_x_*) curves similarly indicated a higher and more sustained reproductive output in *T. harzianum*, with the Mixture showing intermediate performance (Figure 2). Overall, these patterns explain the higher net reproductive rate (*R*_0_) and intrinsic rate of increase (*r*) observed in the *T. harzianum* treatment.

**Figure 3:**
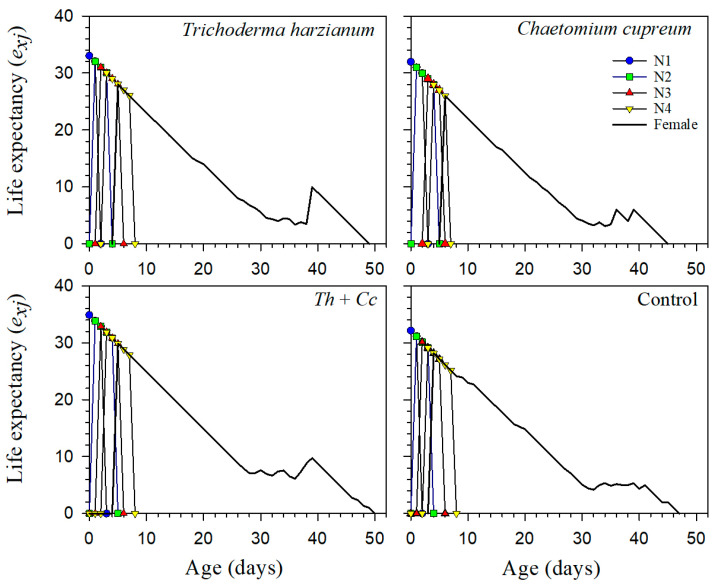
Age-stage life expectancy (*e_xj_*) of *Myzus persicae* feeding on pepper plants treated with *Trichoderma harzianum* (*Th*), *Chaetomium cupreum* (*Cc*), their Mixture (*Th + Cc*), and Control (C).

**Figure 4:**
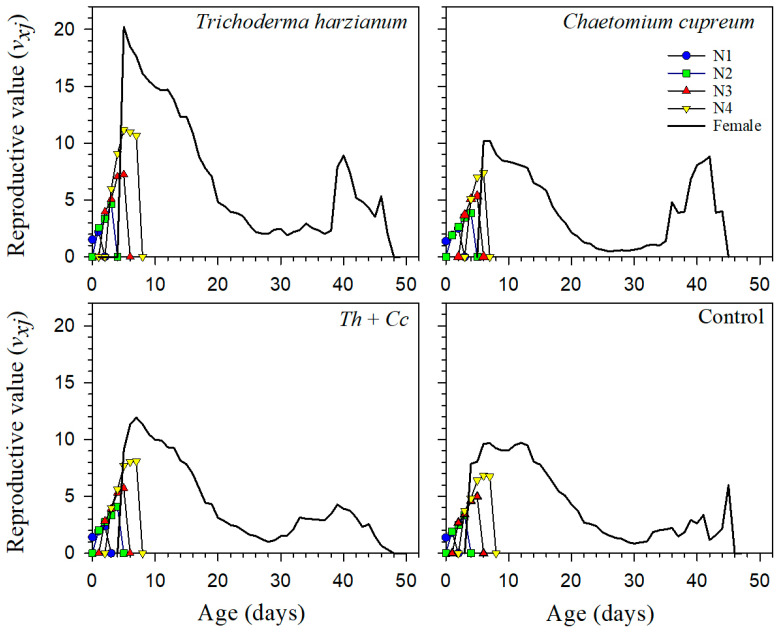
Age-stage specific reproductive value (*v_xj_*) of *Myzus persicae* feeding on pepper plants treated with *Trichoderma harzianum* (*Th*), *Chaetomium cupreum* (*Cc*), their Mixture (*Th + Cc*), and Control (C).

Reproductive value (*v_xj_*) and life expectancy (*e_xj_*) curves further supported these differences among treatments (Figure 3 and Figure 4). In the *T. harzianum* treatment, individuals maintained higher reproductive value during the adult stage and a longer expected remaining lifespan, indicating prolonged contribution to population growth. The Mixture again showed intermediate patterns, whereas the Control and *C. cupreum* treatments showed a more compressed period of high reproductive value and shorter life expectancy.

Together, these demographic patterns indicate that *T. harzianum* improved aphid performance by extending adult longevity and sustaining reproduction, which explains the higher intrinsic rate of increase (*r* = 0.416 d^−1^), shorter doubling time (*DT* = 1.668 days), and greater population growth potential relative to the other treatments.

### 3.3. Population Projection and Growth Dynamics

Population projections (60 days, initiated with 10 newly born nymphs) demonstrated exponential divergence among treatments (Table 4). *T. harzianum*-treated populations reached ~232 billion individuals by day 60, approximately 380-fold greater than controls (611 million). Mixture populations were intermediate (~2.7 billion), while *C. cupreum* (1.03 billion) resembled controls. Stage-structured projections showed that first-instar nymphs consistently dominated (>40% of total) across all treatments, reflecting rapid development and continuous parthenogenetic reproduction typical of *M. persicae*. These projections translate the observed demographic differences into long-term population consequences relevant to pest management. These projections assume unlimited resources and stable conditions. In reality, plant quality degradation, competition, natural enemies, and resource limits would prevent reaching these numbers. However, the projections are useful for comparing relative growth potential and predicting when intervention might be needed.

## 4. Discussion

It was examined how two endophytic fungi (*T. harzianum* and *C. cupreum*) influenced the life history and population dynamics of *M. persicae* on pepper. Contrary to our initial expectation of a biocontrol benefit, *T. harzianum* markedly increased aphid performance, whereas *C. cupreum* had little effect. These results highlight the context-dependence of plant–microbe–herbivore interactions and the need to evaluate beneficial microbes against all key pests in a cropping system.

*T. harzianum* application produced the highest aphid population growth rate, with an intrinsic rate of increase (*r* = 0.416 d^−1^) that significantly exceeded the Control (*r* = 0.315 d^−1^). This increase resulted from dramatically higher fecundity (87.67 vs. 42.90 offspring per individual in Control) combined with extended adult longevity. Population projections showed that *T. harzianum* could result in a population ~380 times larger than C after 60 days, with major implications for aphid management.

These findings are consistent with recent reports of beneficial fungi inadvertently increasing herbivore populations. A study examining *Trichoderma* and *Funneliformis* interactions with *M. persicae* on Mazamort pepper variety reported that *T. asperellum* significantly increased aphid population dynamics (*r* = 0.426 d^−1^ vs. Control 0.386 d^−1^), likely through alterations in plant nutrient composition and hormonal balance that enhanced plant suitability as a host [18]. Interestingly, in the same study, *T. harzianum* showed only a slight, non-significant reduction in aphid populations, demonstrating that even different species within the same genus can produce divergent outcomes. However, other studies have shown contrasting results with *T. harzianum* reducing aphid populations in a cultivar-specific manner. A study conducted on Kasırga F1 pepper variety demonstrated that *T. harzianum* significantly reduced the intrinsic rate of increase of *M. persicae* (*r* = 0.3321 d^−1^) compared to Control (*r* = 0.37 d^−1^), with *T. viride* showing intermediate effects (*r* = 0.3462 d^−1^) [17]. This study further supports the notion that the effects of Trichoderma species on aphid performance are highly dependent on both fungal strain and host plant cultivar, with different pepper varieties eliciting divergent responses even within the same pest-fungus-plant system. In stark contrast, another recent study on the Demre pepper cultivar found that *T. harzianum* had no statistically significant effect on any major population parameters of *M. persicae* (*r* = 0.3148 d^−1^ Control vs. 0.3304 d^−1^ *T. harzianum*, *p* = 0.09), with only the mean generation time showing a significant difference [16]. Additionally, A researcher reported intrinsic rates of increase ranging from 0.193 to 0.248 d^−1^ on different Charleston pepper cultivars, which are lower than the values observed in our Control group (0.315 d^−1^), highlighting the significant influence of host plant genotype on aphid demography [29]. Similarly, Rasool et al. [13] found that *Metarhizium brunneum* seed inoculation unexpectedly increased aphid populations on both wheat and bean plants, an effect attributed to changes in plant secondary metabolite profiles rather than endophytic colonization capacity per se.

*T. harzianum* may have improved aphid performance by altering plant physiology and nutrition, though we cannot confirm this without biochemical data. This fungus improves nitrogen uptake, solubilizes phosphates, and produces siderophores that mobilize micronutrients [10]. If these happened here, higher nitrogen, phosphorus, and micronutrient levels in plant tissues could have benefited nitrogen-limited phloem feeders like aphids [10]. Improved phloem nutritional quality could potentially explain the enhanced aphid growth and reproduction observed, analogous to fertilization effects reported in previous studies [30], though direct measurement of phloem composition was not conducted and would be necessary to confirm this hypothesis.

Furthermore, *Trichoderma* colonization can induce metabolic reprogramming in plants, affecting the tricarboxylic acid cycle and hexose monophosphate pathway, which can alter the concentration and composition of soluble sugars and amino acids in plant tissues [31]. If such changes occurred, any increase in amino acid availability could have been particularly beneficial for aphid reproduction. The longer adult longevity observed in our study (26.90 days for *T. harzianum* vs. 25.93 days for Control) suggests that the nutritional quality of the host plant was sufficient to support extended aphid survival and sustained reproductive output.

*T. harzianum* can also induce plant defenses, activating salicylic and jasmonic acid pathways [32], but defense induction doesn’t always reduce herbivore performance, especially for adapted specialists like M. persicae that tolerate many plant defensive compounds [14]. Without measuring defense compounds, the balance between nutrition and defense effects remains unclear. All plants appeared healthy despite large aphid differences. However, plant growth and yield were not measured. Whether the 2-fold aphid increase on *T. harzianum* plants causes economic damage is unknown. Future studies should measure both aphid populations and plant performance to see if fungal benefits outweigh pest problems.

In contrast to *T. harzianum*, *C. cupreum* treatment showed relatively limited effects on aphid life table parameters. While some individual nymphal instar durations differed from the Control, the total preadult developmental time, fecundity (38.00 offspring), and population growth rate (*r* = 0.323 d^−1^) were not significantly different from the Control. These results suggest that *C. cupreum* colonization had minimal impact on plant nutritional quality or defensive chemistry relevant to aphid performance under our experimental conditions.

The lack of strong effects could be attributed to several factors. First, *C. cupreum* may be less efficient at colonizing pepper roots or may produce lower titers of bioactive metabolites compared to *T. harzianum*. Second, the secondary metabolites produced by *Chaetomium* species, such as chaetoglobosins and azaphilones, are primarily antifungal and may have limited direct or plant-mediated effects on aphids [33]. Third, *C. cupreum* may have different effects on plant physiology that neither strongly enhance nor inhibit plant quality for aphids.

While *Chaetomium* species have demonstrated effectiveness against some insect pests, including cotton aphids (*Aphis gossypii*) and beet armyworms (*Spodoptera exigua*) in previous studies, these effects appear to be pest- and context-specific. Our findings suggest that *C. cupreum* may not be an effective biocontrol agent for *M. persicae* on pepper, at least not through endophytic colonization-mediated plant resistance.

The limited effects of *C. cupreum* could have several explanations, though without direct measurements, these remain speculative. First, *C. cupreum* may have colonized pepper roots less efficiently than *T. harzianum*, or produced lower titers of bioactive metabolites, though colonization levels were not assessed. Second, *Chaetomium* metabolites are primarily antifungal [33] and may have limited effects on phloem-feeding aphids. Third, *C. cupreum* may alter plant physiology in ways that neither strongly enhance nor inhibit aphid performance, resulting in the neutral response observed. *C. cupreum* appears ineffective against *M. persicae* on pepper in this system.

The Mixture treatment produced intermediate effects, with population parameters between *T. harzianum* and Control. Extended longevity in this treatment suggests possible interactions between the two fungi affecting plant-aphid dynamics

The intermediate Mixture effects suggest the fungi interacted in the rhizosphere or plant tissues. Competition likely reduced colonization efficiency or metabolite production, weakening *T. harzianum*’s growth-promoting effects and resulting in intermediate plant changes and aphid performance.

Recent studies examining interactions between different beneficial fungi have revealed complex outcomes. For example, research on *Epichloë* endophytes and arbuscular mycorrhizal (AM) fungi showed that AM fungi presence reversed the negative effects of endophytes on aphids by reducing alkaloid production, demonstrating that multi-species fungal associations can dramatically alter plant-herbivore interactions [34]. Similar antagonistic effects between endophytes have been reported, where the presence of one fungus reduces the efficacy or colonization of another. Further research examining the colonization success and metabolite profiles of both fungi in Mixture treatments would help elucidate the mechanisms underlying these intermediate effects.

The results have important implications for using *Trichoderma* in integrated pest management, especially where aphids are major pests. Although *T. harzianum* is widely used for disease suppression and growth promotion, it can worsen aphid problems by enhancing population growth. Beneficial microorganisms should be evaluated for their effects on all key pests, not just pathogens, before widespread use.

The concept of unintended consequences in biological control is not new, but our study adds to a growing body of evidence showing that plant-associated beneficial microorganisms can have complex, sometimes counterproductive effects on pest populations. Previous field studies with *T. harzianum* T22 reported reduced aphid abundance in some cases, possibly due to enhanced attraction of natural enemies through induced volatile organic compound (VOC) production [35]. However, such tri-trophic effects may be context-dependent and may not occur uniformly across all cropping systems or environmental conditions.

From an IPM perspective, our results suggest that *Trichoderma*-based products should be screened for plant-mediated effects on aphids before field use. Where aphids are an important pest, *Trichoderma* applications should be integrated with complementary tactics (e.g., conservation of natural enemies, reflective mulches, and selective insecticides when necessary) and accompanied by careful monitoring, especially early in the season when plant growth promotion may be strongest.

Age-stage, two-sex life table analysis quantified population-level treatment effects. Reproductive value (*v_xj_*) peaked earlier and higher in *T. harzianum* aphids, and older individuals continued contributing substantially to population growth, a pattern absent in Control and *C. cupreum* treatments, where reproduction was temporally compressed. Life expectancy (*e_xj_*) similarly showed prolonged survival potential in *T. harzianum* aphids.

The population projections revealed the exponential nature of aphid population growth under favorable conditions. The 380-fold increase in projected population size after 60 days for *T. harzianum* compared to Control illustrates how even moderate changes in individual fecundity and survival can translate into dramatic differences at the population level due to the multiplicative nature of population growth in parthenogenetic aphids. This modeling approach is particularly valuable for predicting long-term outcomes and economic thresholds, helping growers anticipate when intervention may be necessary. The 60-day projections are theoretical. The actual numbers (380-fold increase) wouldn’t occur in the field due to plant degradation and other limits. But the relative differences help predict the timing of Control needs.

The stage-specific population dynamics showed that first instar nymphs consistently comprised the largest proportion of the population across all treatments, reflecting the rapid developmental progression characteristic of *M. persicae*. The maintenance of consistent stage structure across treatments despite differences in population growth rates suggests that the fundamental aphid life cycle was not disrupted, but rather accelerated or enhanced by the *T. harzianum* treatment. The utility of the age-stage, two-sex life table in revealing complex demographic responses has been demonstrated in our previous studies on diverse taxa, including *Pseudaulacaspis pentagona* [36] and *M. persicae* on different cultivars [1].

The enhancement of aphid populations by beneficial fungi also raises questions about cascading effects on higher trophic levels. Natural enemies of aphids, including parasitoids and predators, may benefit from higher aphid densities, potentially leading to enhanced biological control over time. However, this would require that natural enemy populations can respond quickly enough to prevent aphid-induced crop damage. Field studies incorporating natural enemy dynamics would be valuable for understanding these complex interactions under realistic conditions.

Furthermore, the vector capacity of *M. persicae* means that even transient increases in aphid populations could lead to increased virus transmission rates, with serious consequences for crop health and yield. The interaction between fungal inoculants, aphid populations, and virus epidemiology warrants careful investigation, particularly for crops like pepper that are susceptible to numerous aphid-transmitted viruses.

Several limitations of this study should be acknowledged. Most importantly, fungal colonization of plant tissues or fungal persistence in the soil/rhizosphere was not verified at the time of aphid introduction. Therefore, it cannot be confirmed whether the observed effects resulted from active fungal colonization, rhizosphere activity, or residual effects from initial fungal presence. First, experiments were conducted under controlled laboratory conditions with a single pepper cultivar. Field conditions, with variable environmental factors, plant genetic diversity, and the presence of natural enemies, may produce different outcomes. Second, fungal colonization levels and plant biochemical changes were not directly measured, which would have provided mechanistic insights into the observed effects.

In this study, fungal colonization or plant biochemical changes (phloem nutrients, secondary metabolites, defense compounds) were not directly measured. Aphid preference experiments were also not conducted. Future studies should include these measurements to determine whether fungal-colonized plants attract aphids and whether the effects are due to improved nutrition, altered chemistry, or both.

## 5. Conclusions

This study advances understanding of multitrophic interactions in agriculture. Plant-endophyte–herbivore relationships are critical but often overlooked in pest dynamics. Most research examines direct fungal effects or induced resistance against chewing herbivores, while effects on phloem feeders through nutrition changes need more study.

*Trichoderma harzianum* and *C. cupreum* produced contrasting effects on *M. persicae* populations. *C. cupreum* had minimal impact, but *T. harzianum* substantially increased aphid growth via enhanced fecundity and longevity, apparently through improved plant nutrition. These results challenge assumptions about universally beneficial effects of growth-promoting fungi and underscore the need for thorough ecological screening before deploying microorganisms in IPM programs.

The results illustrate a key principle in biological control: introducing any organism, even beneficial ones, can have unexpected effects on the broader community. The strain-specific and cultivar-specific responses observed indicate that fungal inoculants must be carefully screened in systems where aphids or other phloem-feeders are important pests.

Future selection of endophytic fungi should consider not only disease suppression and growth promotion, but also indirect effects on herbivores. An integrated approach using multiple biocontrol agents, cultural practices, and compatible pesticides may be needed to capture the benefits of beneficial fungi while avoiding negative consequences. Understanding the mechanisms behind these plant-mediated effects will be essential for developing effective biological control strategies.

## Figures and Tables

**Table 1:** Population parameters, their definitions, and equations used in their calculations.

Parameter	Definition and Equations
*s_xj_*	The probability that a neonate will survive to age *x* and stage *j* [20]. It can be calculated as: $s_{xj}=\frac{n_{xj}}{n_{01}}$ [27], where *n_xj_* is the number of individuals survive to age *x* and stage *j*, *n*_01_ is the number of newborn offspring used at the beginning of life table study.
*m_x_*	The mean number of offspring produced by individuals at age *x*. It is calculated as: $m_{x}=\left(\sum_{j=1}^{m}s_{xj}f_{xj}\right)/\left(\sum_{j=1}^{m}s_{xj}\right)$ [20], where m is the number of stages.
*r*	The population growth rate as time approaches infinity and population reaches the stable age-stage distribution (SASD). The population size will increase at the rate of *e^r^* per time unit. It is calculated by using the Euler-Lotka equation with age indexed from 0 [20,22]: $\sum_{x=0}^{\infty }\left(e^{-r\left(x+1\right)}\sum_{j=1}^{m}s_{xj}f_{xj}\right)=\sum_{x=0}^{\infty }e^{-r\left(x+1\right)}l_{x}m_{x}=1$
*λ*	Finite rate of increase (*λ*): The population growth rate as time approaches infinity and population reaches the stable age-stage distribution. The population size will increase at the rate of *λ* per time unit: $\lambda =e^{r}$
*R*_0_	The total number of offspring that an average individual (including females, males, and those died in immature stage) can produce during its lifetime. It is calculated as: $R_{0}=\sum_{x=0}^{\infty }\sum_{j=1}^{m}s_{xj}f_{xj}=\sum_{x=0}^{\infty }l_{x}m_{x}$ [20].
*T*	It is the period that a population requires to increase to *R*_0_-fold of its size as time approaches infinity and the population settles down to a stable age-stage distribution. $T=\frac{\ln R_{0}}{r}=\frac{\ln R_{0}}{\ln\lambda }$

**Table 2:** Development, longevity, fecundity and oviposition periods of *Myzus persicae* on different treatments (mean ± SE).

Parameters	*Trichoderma harzianum* (*n* = 30)	*Chaetomium cupreum* (*n* = 30)	*T.h.* + *C.c.* (Mix) (*n* = 30)	Control (*n* = 30)
N1 (days)	1.73 ± 0.08 ab	1.93 ± 0.08 a	1.70 ± 0.10 ab	1.50 ± 0.09 b
N2 (days)	1.07 ± 0.05 a	1.23 ± 0.08 a	1.10 ± 0.07 a	1.13 ± 0.06 a
N3 (days)	1.57 ± 0.11 ab	1.57 ± 0.09 a	1.30 ± 0.08 b	1.43 ± 0.09 ab
N4 (days)	1.77 ± 0.13 ab	1.47 ± 0.09 b	2.03 ± 0.14 a	2.13 ± 0.15 a
Preadult time (d)	6.13 ± 0.12 a	6.20 ± 0.07 a	6.13 ± 0.11 a	6.20 ± 0.14 a
Adult Longevity (d)	26.90 ± 1.03 ab	25.77 ± 0.94 b	28.73 ± 1.15 a	25.93 ± 1.53 ab
Total Longevity (d)	33.03 ± 1.03 ab	31.97 ± 0.92 b	34.87 ± 1.14 a	32.13 ± 1.47 ab
TPRP (d)	6.13 ± 0.12 a	6.20 ± 0.07 a	6.13 ± 0.11 a	6.20 ± 0.14 a
Oviposition (d)	18.00 ± 1.48 ab	16.00 ± 1.33 b	21.00 ± 1.64 a	19.00 ± 1.61 a
Fecundity (nymphs)	87.67 ± 7.12 a	38.00 ± 2.90 c	51.27 ± 3.38 b	42.90 ± 3.44 bc

N1–N4: nymphal instars 1–4 (days); TPRP: total pre-reproductive period (days); different letters in the same row indicate significant differences among treatments (*p* < 0.05, paired bootstrap test with 100,000 resampling). *n* = 30 aphids per treatment.

**Table 3:** Population parameters of *Myzus persicae* on pepper plant treated with *Trichoderma harzianum*, *Chaetomium cupreum* and their Mixture.

Parameters	*Trichoderma harzianum* (*n* = 30)	*Chaetomium cupreum* (*n* = 30)	*T.h.* + *C.c.* (Mix) (*n* = 30)	Control (*n* = 30)
Intrinsic rate of increase (*r)* (d^−1^)	0.42 ± 0.01 a	0.32 ± 0.01 bc	0.34 ± 0.01 b	0.32 ± 0.01 c
Finite rate of increase (*λ*) (d^−1^)	1.52 ± 0.02 a	1.38 ± 0.01 bc	1.41 ± 0.01 b	1.37 ± 0.01 c
Net reproductive rate (*R*_0_)	87.67 ± 7.12 a	38.00 ± 2.90 c	51.27 ± 3.38 b	42.90 ± 3.44 bc
Mean generation time (*T*) (d)	10.76 ± 0.29 b	11.25 ± 0.22 ab	11.58 ± 0.25 a	11.94 ± 0.28 a
Gross reproductive rate (*GRR*)	116.01 ± 15.46 a	59.13 ± 11.63 b	64.76 ± 4.75 b	59.10 ± 6.43 b
Doubling time (*DT*) (d)	1.67 ± 0.05 c	2.14 ± 0.04 ab	2.04 ± 0.04 b	2.20 ± 0.06 a

Different letters in the same row indicate significant differences among treatments (*p* < 0.05, paired bootstrap test with 100,000 resampling). *n* = 30 aphids per treatment.

**Table 4:** Projection of population size after 60 days under each treatment.

Treatments	N1	N2	N3	N4	Female	Total Population
Control	225.84 M *	115.53 M	94.48 M	87.46 M	87.61 M	610.93 M
*Trichoderma harzianum*	116.85 B **	40.86 B	33.31 B	21.94 B	18.83 B	231.78 B
*Chaetomium cupreum*	473.09 M	184.51 M	148.20 M	86.15 M	139.24 M	1.03 B
*T.h. + C.c.* (Mix.)	1.16 B	472.88 M	367.13 M	353.09 M	340.94 M	2.69 B

* M: Million; ** B: Billion

## Data Availability

The original contributions presented in this study are included in the article. Further inquiries can be directed to the corresponding author.
